# Psychological and Physical Co-Morbidity among Urban South African Women 

**DOI:** 10.1371/journal.pone.0078803

**Published:** 2013-10-25

**Authors:** Emily Mendenhall, Linda M. Richter, Alan Stein, Shane A. Norris

**Affiliations:** 1 Medical Research Council/Wits Developmental Pathways for Health Research Unit, University of the Witwatersrand, Johannesburg, South Africa; 2 Science, Technology, and International Affairs Program, Walsh School of Foreign Service, Georgetown University, Washington, District of Columbia, United States of America; 3 Human Sciences Research Council, Durban, South Africa; 4 Oxford University, Oxford, United Kingdom; Wits Reproductive Health and HIV Institute, South Africa

## Abstract

**Objectives:**

There is substantial evidence for the links between poverty and both physical and mental health; but limited research on the relationship of physical and mental health problems exists in low- and middle-income countries. The objective of this paper is to evaluate the prevalence and co-morbidity of psychological distress among women with common physical diseases in a socio-economically disadvantaged urban area of South Africa.

**Methods:**

Women enrolled in the Birth to twenty (Bt20) cohort study were evaluated for this paper. Bt20 was founded in 1990 and has followed more than 3,000 children and their caregivers since birth; this study evaluates the health of the caregivers (average age 44) of these children. Psychological distress was evaluated by administering the General Health Questionnaire (GHQ-28) and we evaluated the presence of physical disease by self-report.

**Results:**

Forty percent of the sample presented with psychological distress using the GHQ scoring method. More than half of the women who reported a history of a physical disease, including diabetes, heart attack, asthma, arthritis, osteoporosis, epilepsy, and tuberculosis, reported psychological disorder. Presence of one physical disease was not associated with increased rates of psychological distress. However, women who reported two diseases had increased rates of psychological symptoms, and this upward trend continued with each additional physical disease reported (measured to five).

**Conclusions:**

These data indicate high prevalence rates of co-morbid psychological distress among women with physical disease. This argues for the need of greater mental health support for women living with physical diseases.

## Introduction

The co-occurrence of mental health problems and communicable diseases, such as HIV [1], and non-communicable diseases (NCDs), such as type 2 diabetes [2], present major challenges for public health systems in low- and middle-income countries (LMICs). In Sub-Saharan Africa HIV contributes to a major burden of disease; for example, in South Africa, which is among the most affected countries, the rates among men are 32.9% and women are 46.3% [3]. But in the past decade there have been steady increases of NCDs in South Africa, such as diabetes and heart disease [2], as well as mental disorders [4]. Yet little is known about the co-morbidity of mental and physical health conditions in South African populations or sub-Saharan Africa more generally. 

Mental and behavioral disorders comprise 7.4 percent of the burden of disease globally [4]. Both major depressive disorder and anxiety contribute to a substantial portion of the total burden of disease in southern sub-Saharan Africa, as do social problems such as interpersonal violence [4]. Very few population-level estimates of common mental disorders exist in South Africa, but available studies indicate that common mental disorders, including depression and anxiety, are higher in urban [5] versus rural areas [6] and among women compared to men [7]. Mental distress also is higher among people with diabetes and hypertension [8] and HIV and AIDS [9]. 

People with physical disease have increased risk for mental health problems [10], and premature disability and death as a result of those physical diseases are associated with poor access to health care, particularly among impoverished populations [11]. This is particularly true in low and middle-income countries where the social and economic conditions of poverty are strongly linked with mental distress [10,12,13]. In a recent systematic review, Lund et al found that more than three-quarters of studies reveal a positive association between mental health and a range of poverty indicators, from education, food insecurity, and housing to social class, socio-economic status, and financial stress [12]. For example, The South African Stress and Health (SASH) survey reported that low education levels and low socioeconomic status are associated with increased major depression [7]. The presence of a physical illness can compound further the effects of socio-economic adversity on mental distress [10].

This study evaluates the prevalence of psychological distress and the co-occurrence of psychological distress with twelve physical diseases in urban South African adult women. We evaluate psychological distress according to the General Health Questionnaire (GHQ-28), which provides a composite measure of psychological symptoms by bringing together symptoms that are somatic, anxious, social, and depressive. In addition, we evaluate the relative importance of compounded effects of having more than one physical disease on psychological symptoms across the sample. 

## Methods

### Participants

Birth to Twenty (Bt20) is both the largest and longest running longitudinal birth cohort study of child health and development in Africa [14]. The Bt20 cohort began in 1989 with pilot studies to test the feasibility of a long-term follow-up of mothers and their children for the study of their health and wellbeing [15]. Women were enrolled in their second and third trimester of pregnancy through public health facilities and interviewed regarding their health, social history, and current circumstances. Singleton children (*n* = 3,273) born between April and June 1990 and resident for at least 6 months in the municipal area of Soweto-Johannesburg were enrolled into the birth cohort and have now been followed up 22 times between birth and 23 years of age. Attrition over two decades has been comparatively low (30%), mostly occurring during infancy and early childhood, and approximately 2,300 children and their families remain in contact with the study [15]. The cohort is roughly representative of the demographic parameters of urban South Africa. 

During an annual data collection wave (2007-2008), mothers and caregivers of children in the Bt20 cohort were invited to visit a data collection facility either at Chris Hani Baragwanath Hospital in Soweto or the University of the Witwatersrand Medical School in Johannesburg. Of those invited, 1,743 were eligible for inclusion in this study as being caregivers of Bt20 children and willing to participate. During this data collection wave we administered the General Health Questionnaire (GHQ-28) and a self-report check-list of physical diseases, including type 2 diabetes, high blood pressure, heart attack, stroke, elevated cholesterol, emphysema, asthma, arthritis, osteoporosis, epilepsy, tuberculosis, and cancer. Ethical approval was provided by the University of the Witwatersrand Human Ethics Committee (Reference number M010556).

### General Health Questionnaire (GHQ-28)

The General Health Questionnaire (GHQ-28) consists of four 7-item scales: somatic symptoms, anxiety and insomnia, social dysfunction, and severe depression. It allows for mental health assessment on these four dimensions and an overall mental health score that represents psychological distress, or what other studies have coined “psychiatric caseness” [16]. The structure of all questions is the same. The patient is asked to assess changes in her mood, feelings, and behaviors in the past four weeks. The patient evaluates their occurrence on a 4-point scale. The scale points are: “less than usual”, “no more than usual”, “rather more than usual”, “much more than usual”. The standard scoring method recommended by Goldberg (1979) for case identification is called “GHQ method”. Scores for the first two types of answers are “0” (positive) and for the two others – “1” (negative) [16]. The total responses are then summed. The scoring method maintained a theoretical range of global score (from 0 to 28 points). 

### Analyses

The GHQ was evaluated using the recommended cut-off point of 4/5 [16] and we generated descriptive statistics of psychological symptoms reported according to the four categories of the GHQ: somatic symptoms, anxiety and insomnia, social dysfunction, and major depression. We then evaluated the co-occurrence of psychological distress with reports of physical disease using the chi-square statistic. Finally, we employed a stepwise approach to evaluate the impact of reporting more than one physical disease on psychological symptoms. 

## Results

Three-quarters of the women participating in this study were 40 years of age or older, and more than half was between 40 and 50 years of age (see table 1). Eighty-four percent of the sample self-identified as Black ethnicity and the rest identified as White, Coloured, or Indian. Sixty percent of the sample completed twelve or more years of schooling, and 40 percent did not reach this level of completion, of varying degrees. While study participants were distributed across social class, three-quarters of the sample were in the lowest three of six social classes. Forty percent of the women reported symptoms of psychological distress according to the GHQ-28. 

**Table 1:** Sample Characteristics.

**Variable**	**Total (n, %)**
Age	
(<40 years)	480 (27.5%)
(40-50 years)	978 (56%)
(>50 years)	280 (16%)
Missing	5 (<0.5%)
Ethnicity	
Black (n,%)	1,464 (84%)
White, Indian, or Coloured (n,%)	279 (16%)
Education	
<12 yrs (n=637) (n,%)	637 (36.5%)
12+ yrs (n=1,042) (n,%)	1,042 (60%)
Missing	64 (<0.5%)
Social Class Distribution	
First Quintile (Lowest)	306 (17.5%)
Second Quintile	372 (21%)
Third Quintile	630 (36%)
Fourth Quintile	184 (10.5%)
Fifth Quintile	200 (11.5%)
Sixth Quintile (Highest)	22 (1%)
Missing	29 (1.5%)
Psychological Distress	
None	1,045 (60%)
GHQ-28≥5	698 (40%)

Table 2 shows the distribution of psychological symptoms reported across the sample of women. We found generally high levels of reporting symptoms, including somatic symptoms, anxiety and insomnia, social dysfunction, and severe depression. However, somatic symptoms and anxiety and insomnia demonstrated particularly high levels of symptoms. Although high, fewer women reported symptoms of social dysfunction and severe depression.

**Table 2:** Distribution of Psychological Symptoms.

	**Prevalence** (n, %)
**Somatic Symptoms**	
Been feeling perfectly well and in good health?	361 (21%)
Been feeling in need of a good tonic?	362 (21%)
Been feeling run down and out of sorts?	385 (22%)
Felt that you are ill?	412 (24%)
Been getting any pains in your head?	512 (29%)
Been getting a feeling of tightness or pressure in your head?	426 (24%)
Been having hot or cold spells?	434 (25%)
Summary Score (mean±SD)	1.66 ± 2.23
**Anxiety and Insomnia**	
Lost much sleep over worry?	535 (31%)
Had difficulty in staying asleep once you are fall off “to sleep”?	483 (28%)
Felt constantly under strain?	474 (27%)
Been getting edgy and bad tempered?	461 (26%)
Been getting scared or panicky for no good reason?	378 (22%)
Found everything getting on top of you?	571 (33%)
Been feeling nervous and strung-up all the time?	414 (24%)
Summary Score (mean±SD)	1.90 ± 2.37
**Social Dysfunction**	
Been managing to keep yourself busy and occupied?	109 (6%)
Been taking longer to do the things you do?	246 (14%)
Felt on the whole you were doing things well?	128 (7%)
Been satisfied with the way you’ve carried out your task?	136 (8%)
Felt that you are playing a useful part in things?	92 (5%)
Felt capable of making decisions about things?	102 (6%)
Been able to enjoy your normal day-to-day activities?	168 (10%)
Summary Score (mean±SD)	0.56 ± 1.21
**Depression**	
Been thinking of yourself as a worthless person?	199 (11%)
Felt that life is entirely hopeless?	181 (10%)
Felt that life isn’t worth living?	192 (11%)
Thought of the possibility that you might “make away” with yourself?	215 (12%)
Found at times you couldn’t do anything because your nerves were too bad?	246 (14%)
Found yourself wishing you were dead and away from it all?	152 (9%)
Found that the idea of taking your own life kept coming into your mind?	115 (7%)
Summary Score (mean±SD)	0.75 ± 1.53
GHQ Total (mean±SD)	4.87 ± 5.58

Forty-three percent of the women participating in this study that reported any physical disease reported concurrent symptoms of psychological distress according to the GHQ (Table 3), compared to 40 percent of the overall sample that reported psychological symptoms but not physical disease (Table 1). The percentage of those who reported psychological distress was higher among women who also reported a specific physical disease. More than half of the study participants who reported previous diagnosis of diabetes, heart attack, asthma, arthritis, osteoporosis, epilepsy, and tuberculosis concurrently reported psychological symptoms. Of these analyses, chi-square tests demonstrated significant associations for those reporting heart attack, arthritis, epilepsy, and tuberculosis (Table 3). 

**Table 3:** Co-Morbidity of Psychological Distress and Physical Disorders.

	No Psychological Distress (*n*=1,045)		Psychological Distress (*n*=698)		Total (*n*=1,743)		Chi-Square, P-value
	*N*	%	*n*	%	*n*	%	
No Diabetes	550	60.2%	364	39.8%	914	94.1%	2.72, 0.09
Diabetes	28	49.1%	29	50.9%	57	5.9%	
No High Blood Pressure	393	60.5%	257	39.5%	650	66.9%	0.71, 0.40
High Blood Pressure	185	57.6%	136	42.4%	321	33.1%	
No Heart Attack	571	60.2%	377	39.8%	948	97.6%	8.28, 0.00
Heart Attack	7	30.4%	16	69.6%	23	2.4%	
No Stroke	568	59.5%	386	40.5%	954	98.2%	0.00, 0.95
Stroke	10	58.8%	7	41.2%	17	1.8%	
Low Cholesterol	525	59.3%	361	40.7%	886	91.2%	0.31, 0.58
High Cholesterol	53	62.4%	32	37.6%	85	8.8%	
No Emphysema	562	59.7%	379	40.3%	941	96.9%	0.49, 0.48
Emphysema	16	53.3%	14	46.7%	30	3.1%	
No Asthma	555	60.1%	369	39.9%	924	95.2%	2.30, 0.13
Asthma	23	48.9%	24	51.1%	47	4.8%	
No Arthritis	522	61.6%	326	38.4%	848	87.3%	11.5, 0.00
Arthritis	56	45.5%	67	54.5%	123	12.7%	
No Osteoporosis	571	59.8%	384	40.2%	955	98.4%	1.68, 0.20
Osteoporosis	7	43.8%	9	56.3%	16	1.6%	
No epilepsy	577	59.8%	388	40.2%	965	99.4%	4.60, 0.03
Epilepsy	1	16.7%	5	83.3%	6	0.6%	
No Tuberculosis	570	60.0%	380	40.0%	950	97.8%	4.09, 0.04
Tuberculosis	8	38.1%	13	61.9%	21	2.2%	
No Cancer	575	59.4%	393	40.6%	968	99.7%	2.05, 1.15
Cancer	3	100%	0	0.0%	3	0.3%	
No Physical Illness	299	62.2%	182	37.8%	481	49.5%	2.75, 0.10
Any Physical Illness	279	56.9%	211	43.1%	490	50.5%	
Total	598	61.6%	393	40.5%	971		

We found that among those study participants who reported one physical disease, there was no difference in the percentage of those reporting psychological morbidity (37%) compared to whose who reported no physical disease (37.8%) (Table 4). Although, these percentages were slightly higher than the percentage of psychological distress in the overall sample (40%) (Table 1). In contrast, just over half of the women who reported two and/or three physical problems concurrently reported psychological morbidity. This increased among those who reported four (75%) or five (100%) physical diseases. Thus, these data demonstrate that multi-morbidity increases psychological co-morbidity among the women in our sample. 

**Table 4:** Psychological Distress and Multiple Physical Diseases.

	No psychological Distress		Psychological Distress		Total	
	*n*	%	*n*	%	*n*	%
No Physical Disease	299	62.2%	182	37.8%	481	49.5%
1 Physical Disease	189	63%	111	37%	300	30.9%
2 Physical Diseases	64	48.9%	67	51.1%	131	13.4%
3 Physical Diseases	24	48%	26	52%	50	5.14%
4 Physical Diseases	2	25%	6	75%	8	0.82%
5 Physical Diseases	0	0%	1	100%	1	0.10%
Total	578	59.5%	393	40.5%	971	

Pearson chi-square: 17.27p-value: 0.00

## Discussion

 To our knowledge, this is the first study to examine the co-occurrence of psychological and physical morbidity among urban predominantly Black South African women. We highlight the high burden of psychological distress in this population and reveal the relative import of having a physical disease co-occurring with psychological symptoms, a problem that has been highlighted as a major concern in low and middle-income countries [17]. Moreover, our research demonstrates the importance of examining multiple-morbidity of physical diseases as a contributor to psychological distress. There were four primary findings. 

 First, our study shows that 40 percent of the sample of predominantly Black South African women reported psychological symptoms. This high prevalence can be compared to data from another middle-income country, Iran, where the prevalence of psychological distress (using the GHQ-28) was 26 percent in a sample of 19,000 Iranian women [18]. Although comparative GHQ-28 data are unavailable in South African populations, our findings of 40 percent are much higher than that Tomlinson et al findings that 9.7 percent of a nationally representative sample reported a lifetime major depressive episode and 4.9 reported such an episode for the twelve months prior to the interview [7]. This may be due to the fact that our study evaluated psychological distress generally, which captured somatic symptoms, anxiety, and social dysfunction in addition to depression and, as a result, may have a higher reporting of psychological morbidity. It may also be due to the fact that the GHQ-28 is not a diagnostic tool and therefore may capture higher rates of psychological morbidity than did the Tomlinson et al study. Finally, the elevated rates of psychological morbidity in our sample may result from the fact that our sample was largely poor Black women living in an urban area, given that social and economic conditions, such as social inequality and high rates of violence, are strong predictors of increased mental health problems [12]. 

Second, there has been a very strong emphasis in most studies conducted in sub-Saharan Africa broadly and South Africa specifically on depression, while anxiety and somatic disorders have been largely overlooked. This study indicates that the rates of anxiety and somatic symptoms are if anything considerably higher than depression and that simply measuring depression underestimates the extent of psychological morbidity. The Global Burden of Disease 2010 study indicates that both depression and anxiety are major sources of disability in southern Africa [4] but rarely are somatic symptoms measured or considered in these analyses. A recent review documented the strong association between somatic symptoms with depression and anxiety in women, and suggested that somatic symptoms often are associated with social and economic conditions [19]. Moreover, there is an important link between somatic symptoms and physical morbidity. Hence, understanding the burden of anxiety and somatic symptoms is an important area for further study in southern Africa for evaluating not only the burden of psychological disorders at the population level but also how they may be associated with socioeconomic contexts and linked with physical disease. 

Third, self-reporting of non-communicable diseases, including specifically high blood pressure (33.1%), arthritis (12.7%), high cholesterol (8.8%), type 2 diabetes (5.9%), and asthma (4.8%), demonstrated a high burden of physical disease. In contrast, reporting of tuberculosis (2.2%) was extremely low, which may be a reflection of its stigmatization through its association with HIV. While a limitation of this study is that we do not have evidence of self-reporting HIV, we may consider Tb to be a proxy for HIV infection, as it is a common co-infection. Mayosi et al argued that between 2002 and 2005 for women in South Africa between 50 and 64 years of age HIV and tuberculosis should affect one-quarter of the population [2], and this might be higher among the socially and economically marginalized group in our sample. Therefore, this measure may be underreported. Nevertheless, these prevalence rates match the trend of increasing rates of non-communicable diseases in LMICs [2,4]. 

Fourth, we found co-morbidity of psychological distress and physical disease to be higher (37%) than studies of co-morbidity of psychological and physical disorders conducted with the GHQ-28 in high-income countries (33%) [20]. On the one hand, our data demonstrate that specific diseases might cause more psychological symptoms than others, and that having more than one physical disease greatly increases one’s risk of psychological morbidity. For example, among seven of the twelve physical diseases, more than half of those who self-reported the disease had concurrent psychological disorder. Specifically, heart attack, arthritis, epilepsy, and tuberculosis may be the most distressing diseases. Even more, an individual who had had a heart attack may also have diabetes and the co-occurrence of these two diseases may exacerbate the likelihood of psychological problems. On the other hand, it is very likely that the relationship between psychological distress and physical disease is bidirectional, as those with psychological symptoms may be more likely to develop physical diseases, specifically NCDs, and vice versa. Chronic, untreated psychological disorders over the life course have been associated with increased risk for physical diseases, and metabolic conditions in particular [21]. Thus, the data underscore the importance of multi-morbidity on psychological disorder and the bi-directionality of this relationship in LMICs, an area that requires future research. 

This study has a number of limitations. As is the nature of large longitudinal studies in difficult circumstances, there was some variability in the timing of data collection across the year. In addition, we relied on self-reporting of physical diseases and we are busy collating clinical data to verify the self-reported data and to identify those women that may not be aware of a physical disease (i.e. undiagnosed). However, all the data presented in this study was cross-sectional data and therefore is restricted to one psychological assessment. Moreover, we did not evaluate co-occurring mental illness. Further, our data was collected primarily on Black South African women living in an urban residential neighborhood, and this affects the generalizeability to other ethnic groups living in the region. More research is needed to evaluate variation of psychological symptoms across women of different ethnic groups in urban areas as well as rural regions. Nevertheless, this study provides evidence of the need for more research on psychological disorders and co-morbidity with physical diseases in sub-Saharan Africa, and with NCDs in particular.

## Conclusion

This study underscores the importance of examining the co-morbidity of psychological morbidity among people with physical diseases [17]. The results not only demonstrate the presence of co-morbid psychological distress with various physical diseases, but also they indicate that the presence of more than one physical disease, and incremental increases in the reporting of physical diseases, may have a bigger impact on psychological morbidity than one physical disease alone. Policy-makers need to recognize the importance of psychological co-morbidity among people with physical diseases, and the relative impact of physical disease on psychological disorders in LMICs [17] in order to improve mental health and social services for socially and economically marginalized groups who are concurrently the most affected. 
